# Parental education and youth suicidal behaviours: a systematic review and meta-analysis

**DOI:** 10.1017/S204579602200004X

**Published:** 2022-03-30

**Authors:** P. J. Chen, N. Mackes, C. Sacchi, A. J. Lawrence, X. Ma, R. Pollard, M. Matter, C. Morgan, S. Harding, G. Schumann, C. Pariante, M. A. Mehta, G. Montana, C. Nosarti, P. Dazzan

**Affiliations:** 1 Department of Psychological Medicine, Institute of Psychiatry, Psychology, and Neuroscience, King's College London, London, UK; 2 Department of Psychiatry, Chang Gung Memorial Hospital at Taoyuan and Chang Gung University, Taoyuan, Taiwan; 3 Department of Developmental Psychology and Socialisation, University of Padova, Padua, Italy; 4 Health Service & Population Research Department, Institute of Psychiatry, Psychology, and Neuroscience, King's College London, London, UK; 5 Division of Diabetes and Nutritional Sciences, King's College London, London, UK; 6 Biological Psychiatry, Institute of Psychiatry, Psychology and Neuroscience, King's College London, London, UK; 7 Department of Neuroimaging & Psychopharmacology, Centre of Neuroimaging Sciences, King's College London, London, UK; 8 Department of Data Science, University of Warwick, Coventry, UK; 9 Department of Child and Adolescent Psychiatry, Institute of Psychiatry, Psychology, and Neuroscience, King's College London, London, UK; 10 Centre for the Developing Brain, Department of Perinatal Imaging & Health, School of Biomedical Engineering & Imaging Sciences, King's College London, London, UK

**Keywords:** Adolescence, adolescent, country income level, geographical region, parental education, suicide, suicidal attempt, suicidal behaviour, suicidal ideation, socioeconomic status, youth

## Abstract

**Aims:**

Lower parental education has been linked to adverse youth mental health outcomes. However, the relationship between parental education and youth suicidal behaviours remains unclear. We explored the association between parental education and youth suicidal ideation and attempts, and examined whether sociocultural contexts moderate such associations.

**Methods:**

We conducted a systematic review and meta-analysis with a systematic literature search in PubMed, PsycINFO, Medline and Embase from 1900 to December 2020 for studies with participants aged 0–18, and provided quantitative data on the association between parental education and youth suicidal ideation and attempts (death included). Only articles published in English in peer-reviewed journals were considered. Two authors independently assessed eligibility of the articles. One author extracted data [e.g. number of cases and non-cases in each parental education level, effect sizes in forms of odds ratios (ORs) or beta coefficients]. We then calculated pooled ORs using a random-effects model and used moderator analysis to investigate heterogeneity.

**Results:**

We included a total of 59 articles (63 study samples, totalling 2 738 374 subjects) in the meta-analysis. Lower parental education was associated with youth suicidal attempts [OR = 1.12, 95% Confidence Interval (CI) = 1.04–1.21] but not with suicidal ideation (OR = 1.05, 95% CI = 0.98–1.12). Geographical region and country income level moderated the associations. Lower parental education was associated with an increased risk of youth suicidal attempts in Northern America (OR = 1.26, 95% CI = 1.10–1.45), but with a decreased risk in Eastern and South-Eastern Asia (OR = 0.72, 95% CI = 0.54–0.96). An association of lower parental education and increased risk of youth suicidal ideation was present in high- income countries (HICs) (OR = 1.14, 95% CI = 1.05–1.25), and absent in low- and middle-income countries (LMICs) (OR = 0.91, 95% CI = 0.77–1.08).

**Conclusions:**

The association between youth suicidal behaviours and parental education seems to differ across geographical and economical contexts, suggesting that cultural, psychosocial or biological factors may play a role in explaining this association. Although there was high heterogeneity in the studies reviewed, this evidence suggests that the role of familial sociodemographic characteristics in youth suicidality may not be universal. This highlights the need to consider cultural, as well as familial factors in the clinical assessment and management of youth's suicidal behaviours in our increasingly multicultural societies, as well as in developing prevention and intervention strategies for youth suicide.

## Introduction

Suicide is the third leading cause of death among youths worldwide (Chen *et al*., 2020). Suicidal behaviours, including suicidal ideation (thought of killing oneself) and suicidal attempt (non-fatal, self-inflicted destructive acts with explicit or inferred intent to die), are well recognised precursors of suicide death. In fact, evidence suggests that over one-third of youths with suicidal ideation go on to attempt suicide, and suicide rates consistently increase from childhood to adolescence (Cha *et al*., 2018). A greater understanding of the risks associated with suicidal behaviours is needed in order to guide more effective intervention and prevention strategies in context-specific ways (Dervic *et al*., 2006; Yip *et al*., 2015). Identifying these risk factors in this particular age group across different societies is therefore of pressing importance. However, existing studies have been largely limited by the use of relatively small sample sizes and by the evaluation of cohorts mostly collected in a single, high-income country (HIC) (Yip *et al*., 2015).

Family characteristics, along with individual and societal factors, have been shown to contribute to youth suicidal behaviours, and among these, family socioeconomic disadvantage has been suggested to be one of the major risk factors (Aggarwal *et al*., 2017). Family socioeconomic status (SES) is associated with a wide array of exposures, resources and susceptibilities that may impact health (Galobardes *et al*., 2006), and families with lower SES suffer from multiple forms of disadvantage (Reiss *et al*., 2019). Through material hardship, greater parental stress and parental mental health problems and harsher parenting, familial socioeconomic inequalities can contribute to poor mental outcomes on the offspring (Weinberg *et al*., 2019).

Parental education, as one of the most commonly assessed indicators of familial SES, has been widely studied for its relation to youth mental health outcomes, and found to play a role even when other socioeconomic confounders are taken into account (Sonego *et al*., 2013). Furthermore, parental education has been found to have a stronger relationship with child and adolescent mental health compared to other family SES indicators, such as parental unemployment or lower occupational status (Reiss *et al*., 2019). Parental education, specifically reflecting the possession or availability of knowledge, has been noted to affect parenting styles (Carr and Pike, 2012), disciplinary practices (Bøe *et al*., 2014), health investment (Lindeboom *et al*., 2009), home literacy environment (Keshavarz and Baharudin, 2013) and parental school involvement (Padilla-Moledo *et al*., 2016), which have been proposed to independently and/or jointly influence youth mental health outcomes.

When it comes to youth suicidality, there is yet no agreement as to whether and how parental education could be associated with a higher risk. While some studies have reported lower parental education to be a risk factor for youth suicidal behaviours (Dubow *et al*., 1989; Andrews and Lewinsohn, 1992; Evans *et al*., 2004), others have found no association or even a protective role (Gage, 2013; Chang *et al*., 2017). Differences in sociocultural contexts in these studies have been proposed to be contributing to these contradictory findings (Bøe *et al*., 2012). As a result, an effort should be made to further elucidate the role of sociocultural contextual differences in these studies, as this could not only help the interpretation of results, but also highlight potential different mediating pathways through which parental education could be related to the risk of youth suicidal behaviours across the globe. Therefore, we conducted this first systematic review and synthesis of empirical evidence on parental education and youth suicidal behaviours, while taking into account the possible role of sociocultural contexts, as reflected by geographical region and country income level.

The primary goal of this systematic review was to establish whether there is an association between parental education and either youth suicidal ideation or suicidal attempts (including suicide death). Our secondary goal was to determine if geographical region and country income level could potentially moderate any observed association.

## Methods

### Search strategy

We followed the Meta-analyses of Observational Studies in Epidemiology (MOOSE) guidelines (Stroup *et al*., 2000). We conducted a systematic search on PubMed, PsycINFO, Medline and Embase to screen for studies reporting on the association between parental education and youth suicidality. We applied the following search string: (family OR familial OR household OR parental OR caregiver OR guardian OR mother OR maternal OR father OR paternal) AND (education* OR school*) AND (suicid* OR parasuici* OR ‘self-harm’ OR ‘self-injur*’ OR ‘self-poison*’ OR ‘self-cut*’ OR ‘self-destruct*’ OR ‘self-inflict*’) AND (teen OR teenager OR adolescen* OR children OR youngster OR youth). We limited search results to (1) English publications, (2) peer-reviewed journals and (3) published between January 1900 and December 2020. Two authors (P. J. C. and N. M.) independently assessed the eligibility of each study. When eligibility could not be established through titles and abstracts, the authors retrieved the full text. Any discrepancy was resolved through discussion and opinion of a third author (P. D.). The search strategy initially yielded a total of 6091 articles (after de-duplication). The search was later supplemented by a screening of the references of the studies included.

### Inclusion criteria

We included papers that fulfilled the following criteria: (1) education of parents (or parental figures, such as caregivers or household heads) was assessed and reported as a categorical variable, or reported with beta coefficients if education was measured as a continuous variable; (2) youth suicidal behaviour (thoughts/ideations, attempts or deaths) was assessed separately and independently from other constructs (i.e. other risky behaviours or mental disorders) before the age of 18 (included); (3) concrete case number or person-years data in accordance with different parental educational level was provided, or quantitative associations between parental education level and adolescent suicidal behaviour was reported in the forms of odds ratio (OR) or beta coefficients. We excluded studies of youths with autism spectrum disorders, schizophrenia spectrum disorders and intellectual disabilities. For studies that investigated the same population, we chose the larger or, where this was equal, the most recent one. Reviews, meta-analysis, commentaries, editorials and correspondences were not included.

### Study factor

Parental education level, the main study factor, was assessed and reported differently across studies. For the primary analyses, we coded studies according to their treatment of parental education level as a predictor of outcome. For the secondary analyses, we re-categorised parental educational levels into low, middle and high for the purpose of standardisation. Using the International Standard Classification of Education level 3 (ISCED 3; http://uis.unesco.org) as the cut-off point, we categorised an education level below ISCED 3 as low education (i.e. illiteracy, no education, basic or primary education, middle school, lower secondary education or education years below 12); an education level equals to ISCED 3 as middle education (i.e. upper secondary education, high school graduate or education years equal to 12) and an education level above ISCED 3 as high education (i.e. college/university/master/doctoral degree or education years above 12).

### Outcomes

Outcomes of interest were youth suicidal ideation and suicidal attempts (including suicide death). We used the definitions or criteria made to determine positive outcomes in each study. However, studies on youth self-harming behaviours that did not specify whether this had a suicidal intent were excluded from the present review.

### Data extraction

General study characteristics including name of the first author, publication year, country/region where the study was conducted, cohort name, case definition and outcome type were extracted. We also extracted: (1) classifications of parental education; (2) methods of assessment of parental education and youth suicidal behaviour (questionnaire, interview or data-linkage); (3) source of information about suicidal behaviours (adult-report, children-report or data-linkage); (4) timeframe of suicidal behaviour assessment (lifetime or specific timeframe, such as e.g. previous 6–12 months); (5) type of data from which the association was determined (cross-sectional or longitudinal); (6) sample type (community or clinical); (7) female/male participant ratio; (8) study country income level as per The World Bank 2021 data (high or low and middle; https://datahelpdesk.worldbank.org) and (9) study geographical region based on the sustainable development goal indicators, the regional groupings defined under the Standard Country or Area Codes for Statistical Use of the United Nations Statistics Division (sub-Saharan Africa, Northern Africa and Western Asia, Central and Southern Asia, Eastern and South-Eastern Asia, Latin America and the Caribbean, Oceania, Europe and Northern America; https://unstats.un.org/sdgs/indicators/regional-groups). For pooling, we obtained the maximally adjusted estimate of the OR compared with the reference for each education level, and the corresponding 95% confidence interval (CI). If ORs were unavailable, we computed ORs from raw data presented in the original studies. If the study measured parental education in years and reported only beta coefficients, we multiplied the coefficients by 4 (a correction factor chosen to reflect the difference in mean years of education between high- and low-parental education level) to better align the results with the rest of the studies on the same scale. If both maternal and paternal education levels were provided, maternal education level was chosen as representative, as more studies chose maternal education as a proxy for parental education. If the study provided survey year or sex stratification of the youths, the results were analysed separately.

### Risk of bias assessment

We used the Newcastle–Ottawa Quality Assessment Scale for (1) cross-sectional studies, (2) cohort studies and (3) case control studies to assess risk of bias. Information on (1) sample selection, (2) comparability of cohorts and (3) assessment of outcome were collected. For cohort studies, however, we did not include the question about whether follow-up duration was sufficiently long for the outcome to occur, as this was not applicable. As a result, a maximum score of 8, 8 and 9 could be reached for cross-sectional studies, cohort studies and case control studies, respectively. A total score of 0–4 was considered as indicative of high risk of bias; 5–6 of some concern and 7–9 of low risk of bias.

### Data analysis

Random effects meta-analyses with DerSimonian–Laird estimator (DerSimonian and Laird, 1986) were conducted using R (version 4.0.3 GUI 1.73) with the metaphor (Viechtbauer, 2010) and meta (Balduzzi *et al*., 2019) packages to estimate pooled ORs and 95% CI. Suicidal ideation and suicidal attempt/death were treated as separate outcomes and analysed independently. For the primary analysis, we first derived pooled estimates of the association with outcomes of the lowest parental education level against the highest parental education level from each study with the highest level as the reference; if the study treated parental education as a continuous variable or only provided regression coefficients, we used the beta coefficients (corrected as aforementioned if education was measured in years) as the log odds (Szumilas, 2010). We then performed secondary analyses by pooling estimates of the middle parental education level group (equal to ISCED 3) against the high group (above ISCED 3) with the high group as the reference, the low group (below ISCED 3) against the middle group with the middle group as the reference, and the low group against the high group with the high group as the reference. Secondary analyses were designed to reveal more details on whether and how a specific parental educational achievement could be associated with youth suicidal behaviours.

Heterogeneity was assessed by *Q* test and *I*^2^ statistics. An *I*^2^ value of 50% was indicative of moderate heterogeneity, whereas 75% was considered substantial. When heterogeneity was observed in the data, we tested moderating effects by applying mixed-effects models. Geographical region and country income level were selected as moderators of interest. Other potential moderators investigated were sample type, female ratio, study design, outcome assessment methods, outcome assessment subject, timeframe of the assessed outcome and risk of bias. Risk of publication bias was assessed via visual inspection of funnel plots, supplemented by Egger's test (Egger *et al*., 1997).

## Results

**Fig. 1.**
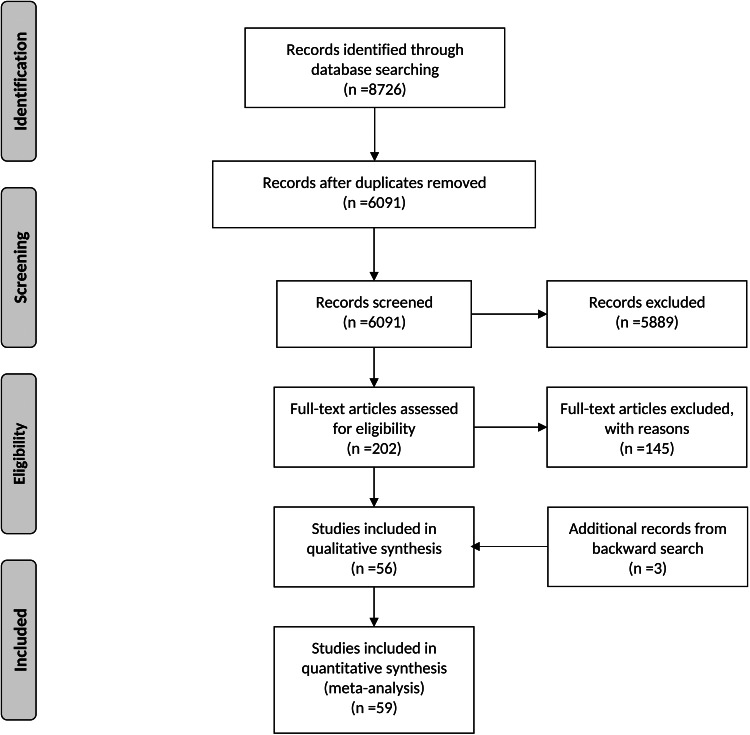
Flow diagram of the present systematic review and meta-analysis.

We identified 8726 articles from PsycINFO, Medline, Embase and PubMed. Of these, 2635 were duplicates and were therefore removed, with 6091 remaining. Further 5889 were later excluded based on titles and abstracts. An additional 145 studies were excluded following screening of full texts. Backward search of the references of the remaining 56 articles resulted in three additional records, leaving a total of 59 articles satisfying the eligibility criteria (Fig. 1).

The 59 articles, published between 1900 and 2020, encompassed 63 eligible study samples, with samples ranging 35 to 2 395 677 individuals, with a total sample size of *n* = 2 738 374. Details of the samples included are presented in Table 1.

**Table 1.** Characteristics of the studies included in the meta-analysis

Authors (year, country/region)	Sample size, *N*	Female sex (%)	Age	Sample	Study type^a^	Parental education		Suicidal behaviour				
						Parent evaluated	Classification	Ideation/attempt	Tool	Assessment	Subject	Timeframe
Abdeen *et al*. (2018, Palestine)	5713	0.7	13	Community	Cross-sectional	M	Below secondary; secondary; above secondary	Both	HBSC-ME	Q	Child	12 months
Alaimo *et al*. (2002, USA)	754	0.52	15–16	Community	Cross-sectional	O	Below high school; high school; above high school	Both	DIS	I	Child	Lifetime
Allen and Goldman-Mellor (2018, USA)	4463	0.49	14.6	Community	Cross-sectional	O	No education; high school; some college; college graduate	Ideation	–	I	Child	12 months
Amit *et al*. (2014, Israel)	620	0.48	14–17	Community	Cross-sectional	M	0–11; 12; 13 or more years	Both	DAWBA	I	Both	4 weeks
Anteghini *et al*. (2001, Brazil)	1960	0.55	13–17	Community	Cross-sectional	O	No more than high school; some college	Both	CAHS	Q	Child	Lifetime
Armağan *et al*. (2020, Turkey)	60	0.93	12–18	Clinical	Cross-sectional	M	Primary school; secondary school; high school	Attempt	–	I	Both	June–December 2017
Asarnow *et al*. (2011, USA)	327	0.7	15.9	Clinical	Cross-sectional	O	At least college graduate	Attempt	K-SADS	I	Both	Lifetime
Assari *et al*. (2020, USA)	3271	0.5	9.5	Community	Cross-sectional	O	Did not complete high school; completed high school	Attempt	K-SADS	I	Adult	Lifetime
Beattie *et al*. (2019, India)	1191	1	13–14	Community	Cross-sectional	O	Illiterate; literate	Ideation	–	Q	Child	2 weeks
Bolat *et al*. (2017, Turkey)	142	0.85	14.5	Clinical	Cross-sectional	M	Years	Attempt	Referral	I	Child	November 2014–November 2015
Borges *et al*. (2008, Mexico)	3005	–	12–17	Community	Cross-sectional	O	None/elementary school; junior high school; high school; university+	Both	WMH-CID-A	I	Child	Lifetime
Bush and Qeadan (2020, USA) sample 1	2661	–	14–17	Community	Cross-sectional	M	Below high school; high school; above or equal to college	Attempt	NM-YRRS	Q	Child	12 months
Bush and Qeadan (2020, USA) sample 2	3473	–	14–17	Community	Cross-sectional	M	Below high school; high school; above or equal to college	Attempt	NM-YRRS	Q	Child	12 months
Bush and Qeadan (2020, USA) sample 3	3117	–	14–17	Community	Cross-sectional	M	Below high school; high school; above or equal to college	Attempt	NM-YRRS	Q	Child	12 months
Chang *et al*. (2017, China)	13 952	0.47	10–18	Community	Cross-sectional	M	Primary school or below; junior middle school; high school or technical secondary school; college or above	Attempt	–	Q	Child	12 months
Chau *et al*. (2013, France)	1559	0.5	13.5	Community	Cross-sectional	O	Low-parental education	Attempt	Kandel Scale	Q	Child	Lifetime
Chen *et al*. (2020, China)	610	0.49	15	Community	Cross-sectional	O	Unspecified	Ideation	BSSI	Q	Child	Lifetime
Chiu *et al*. (2017, Taiwan)	2896	0.5	15	Community	Cross-sectional	M	≤12; >12 years	Ideation	SCL-15	Q	Child	1 week
Cornell and Huang (2016, USA)	47 888	0.51	14–17	Community	Cross-sectional	O	Did not graduate from high school; graduated from a high school; graduated from a 2-year college; completed post-graduate studies	Both	YRBS	Q	Child	12 months
DiLLi *et al*. (2010, Turkey)	136	0.83	13.8	Clinical	Cross-sectional	M	Illiterate; primary school; high school, university	Attempt	SPS	I	Child	November 2005–September 2007
Franić *et al*. (2011, USA)	803	0.5	12	Community	Cross-sectional	M	8; 8–12; >12 years	Ideation	–	Q	Child	Lifetime
Freuchen *et al*. (2012, Norway)	378	–	0–15	Community	Cross-sectional	M	Elementary/secondary school; university	Attempt	Linkage	Linkage	Linkage	Lifetime
Gage (2013, Ethiopia)	2709	1	14.2	Community	Cross-sectional	O	Neither educated; one parent; both parents educated	Both	–	Q	Child	3 months
Haavisto *et al*. (2005, Finland)	2098	0	18	Community	Cross-sectional	O	Not graduated from upper secondary school; graduated from upper secondary school	Both	–	Q	Child	6 months
Kim *et al*. (2019, Korea)	3201	0.45	15.1	Community	Cross-sectional	M	≤6; 7–9; 10–12; ≥13 years	Ideation	–	Q	Child	1 year
King *et al*. (2019, USA)	2104	0.63	15.1	Clinical	Cross-sectional	M	High school graduate or less; some college/technical training; college graduate/professional	Attempt	C-SSRS	Q	Child	June 2015–July 2016
Kokkevi *et al*. (2011, Greece)	46 668	–	14–18	Community	Cross-sectional	O	Primary/unknown; beyond primary	Attempt	–	Q	Child	Lifetime
Kovess-Masfety *et al*. (2015, Europe based, multi-countries)	4491	0.49	8.7	Community	Cross-sectional	M	High school not completed; high school completed; continued after high school	Ideation	DI	Q	Child	Lifetime
Lee and Shin (2017, Korea)	72 435	0.49	12–17	Community	Cross-sectional	M	Below high school graduation; high school graduation; above college graduation; missing	Both	KYRBS	Q	Child	12 months
Leslie *et al*. (2010, USA)	993	0.57	11–15	Community	Cross-sectional	O	Below high school; high school diploma/equivalent; above high school	Attempt	–	I	Child	Lifetime
Liang *et al*. (2014, China)	2131	0.49	13.9	Community	Cross-sectional	M	≤9; >9 years	Attempt	SHQ	Q	Child	1 year
Liu *et al*. (2019, China)	11 831	0.49	15	Community	Cross-sectional	O	Primary school; middle school; high school; professional school; college or above	Both	AHQ	Q	Child	1 year
Liu *et al*. (2005, China)	284	0.4	15.6	Community	Cross-sectional	M	Primary school or less; middle school; high school; college	Attempt	YSR	Q	Child	6 months
Liu and Sun (2005, China)	1920	0.45	13.6	Community	Cross-sectional	M	Illiterate/semi-illiterate; primary school; middle school; high school; college	Ideation	CBCL	Q	Both	6 months
Lu *et al*. (2020, China)	464	0.46	11–17	Community	Cross-sectional	M	Primary school or below; middle school; high school or above	Ideation	SDQ	Q	Child	1 year
Maimon *et al*. (2010, USA)	990	0.52	11–16	Community	Cross-sectional	M	Below high school; some high school; finished high school; above high school; bachelor's degree or more	Attempt	–	I	Child	Lifetime
Mars *et al*. (2014, UK)	4799	0.59	16	Community	Cross-sectional	M	Below O level; O-level; A level; degree	Attempt	CASE	Q	Child	Lifetime
Martin *et al*. (2016, USA)	360	0.28	3–7	Clinical	Cross-sectional	M	Completed high school/GED	Both	DIPA	I	Adult	Lifetime
Min *et al*. (2012, Korea)	676	0.5	6.5	Community	Cross-sectional	O	Both parents college educated; one parent college educated; neither parent college educated	Ideation	BASC-2	Q	Adult	Lifetime
Nock *et al*. (2013, USA)	6483	–	13–18	Community	Cross-sectional	O	Below high school; high school; some college; college graduate	Both	CIDI	I	Child	Lifetime
Oppenheimer *et al*. (2018, USA)	238	0.57	12.2	Community	Longitudinal	O	Above or equal to BA	Ideation	SITBI	I	Both	Lifetime
Paul and Ortin (2019*a*, USA)	1090	0.51	6	Community	Cross-sectional	O	Years	Both	CBCL	I	Adult	6 months
Paul and Ortin (2019*b*, USA)	2958	0.47	9	Community	Cross-sectional	O	Below high school; high school or equivalent; some college or higher	Both	CBCL	I	Adult	6 months
Peter *et al*. (2008, Canada)	1032	0.53	12–15	Community	Cross-sectional	O	Highest level of parental education	Ideation	NLSCY	Q	Child	12 months
Phil and Minde (1995, Canada)	35	1	13–16	Community	Cross-sectional	O	Both parents have 0–6 years; one parent has 0–6 years, the other has 7 or more years; both parents have 7 or more years	Attempt	–	I	Both	Lifetime
Resch *et al*. (2008, Germany)	1681	–	7–17	Community	Cross-sectional	O	Low-parental education	Both	YSR	I	Both	Lifetime
Reyes *et al*. (2011, Puerto Rico)	585	0.53	12–15	Community	Cross-sectional	M	Below high school; completed high school; above high school	Attempt	CAPI	I	Child	12 months
Sabo *et al*. (2005, USA) sample 1	7993	1	14–18	Community	Cross-sectional	M	Years	Both	–	Q	Child	1 year
Sabo *et al*. (2005, USA) sample 2	7825	0	14–18	Community	Cross-sectional	M	Years	Both	–	Q	Child	1 year
Sampasa-Kanyinga and Hamilton (2016, Canada)	4955	0.52	15.2	Community	Cross-sectional	O	High school or less; some college/university; university degree; do not know	Both	YRBS	Q	Child	12 months
Shin *et al*. (2009, Korea)	1857	0.51	13.8	Community	Cross-sectional	M	≤12; >13 years	Both	K-YSR	Q	Child	6 months
Slap *et al*. (2001, USA)	6517	0.5	16	Community	Cross-sectional	O	Below high school or equivalent; no VS; high school or equivalent, or VS; VS or college after high school graduation; college graduate; professional school	Attempt	Likert Scale	I	Child	12 months
Steck *et al*. (2018, Switzerland)	2 395 677	0.49	10–18	Community	Longitudinal	O	Compulsory; secondary; tertiary; not known	Attempt	Linkage	Linkage	Linkage	Lifetime
Toros *et al*. (2004, Turkey)	4143	0.5	11–16	Community	Cross-sectional	M	Years	Attempt	CBDI	Q	Child	Lifetime
Tran *et al*. (2020, Vietnam)	6427	0.54	13–17	Community	Cross-sectional	M	High school and lower; diploma and higher	Ideation	CES-D	Q	Child	12 months
Wang *et al*. (2019, China)	1347	0.48	12.5	Community	Cross-sectional	O	Elementary or less; middle/high school; college or above; not sure	Ideation	–	Q	Child	1 month
Whetstone *et al*. (2007, USA) sample 1	2197	1	10–16	Community	Cross-sectional	O	Below high school; high school graduate; some college or above	Both	YRBS	Q	Child	Lifetime
Whetstone *et al*. (2007, USA) sample 2	2095	0	10–16	Community	Cross-sectional	O	Below high school; high school graduate; some college or above	Both	YRBS	Q	Child	Lifetime
Xiao *et al*. (2020, China)	2898	0.48	14	Community	Cross-sectional	M	Elementary and below; senior high school and above	Ideation	BSSI	Q	Child	Lifetime
Yuen *et al*. (2000, USA)	3327	0.52	–	Community	Cross-sectional	O	Below or equal to high school; some college or more	Attempt	MLES	Q	Child	Lifetime
Zalsman *et al*. (2016, Israel)	957	0.49	14–17	Community	Cross-sectional	M	0–11; 12; 13+ years	Both	DAWBA	I	Child	Lifetime
Zhang *et al*. (2018, China)	16 271	0.48	15.3	Community	Cross-sectional	M	Elementary or below; junior high school; senior high school; college or above	Both	–	Q	Child	12 months
Zubrick *et al*. (2016, Australia)	2653	–	12–17	Community	Cross-sectional	O	Year 10 or below; year 11 or 12; diploma or certificate III/IV; bachelor's degree or higher	Both	YRBS	Q	Child	12 months

AHQ, Adolescent Health Questionnaire; BASC-2, Behaviour Assessment System for Children; BSSI, Beck Scale for Suicidal Ideation; CAHS, Canada Adolescent Health Survey; CAPI, Computer-Assisted Personal Interviewing; C-SSRS, Columbia-Suicide Severity Rating Scale; CASE, Child and Adolescent Self-harm in Europe; CBCL, Child Behaviour Checklist; CBDI, Child Beck Depression Inventory; CES-D, Center for Epidemiologic Studies Depression; CIDI, Composite International Diagnostic Interview; DAWBA, Development And Well-Being Assessment; DI, Dominic Interactive; DIPA, the Diagnostic Infant and Preschool Assessment; DIS, Diagnostic Interview Schedule; HBSC-ME, Health Behaviour in School aged Children in the Middle East study; I, Interview, K-SADS, Kiddie Schedule for Affective Disorders and Schizophrenia; KYRBS, Korean Youth Risk Behaviour Survey; K-YSR, Youth Self Report-Korean version; M, Mother; MLES, Major Life Events Scale; NLSCY, National Longitudinal Survey of Children and Youth; NM-YRRS, New Mexico Youth Risk and Resiliency Survey; O, Other; Q, Questionnaire, SCL-15, Symptom Checklist-15 item version; SDQ, Strengths and Difficulties Questionnaire; SHQ, Self-Harm Questionnaire; SITBI, Self-Injurious Thoughts and Behaviours Interview; SPS, Suicide Probability Scale; WMH-CIDI-A, World Mental Health computer assisted Adolescent version of the Composite International Diagnostic Interview; YRBS, Youth Risk Behaviour Survey; YSR, Youth Self Report.

a ‘Cross-sectional’ type refers to the outcome data used to determine the association in the study was assessed at a single timepoint; ‘longitudinal’ type refers to the outcome data used to determine the association in the study was repeatedly assessed and accumulated during the follow-up period.

The samples were mainly from the community (*k* = 57), with only six studies including clinical populations. Overall, 61 samples estimated the association between parental education and youth suicidal behaviour using outcome data measured at a single time point (cross-sectional), and two samples used cumulative outcome data from repeated assessments obtained during a follow-up period (longitudinal). Most of the samples were from Europe and Northern America (*k* = 34), followed by Eastern and South-Eastern Asia (*k* = 16), Western Asia (*k* = 7), Latin America and the Caribbean (*k* = 3), Central and Southern Asia (*k* = 1), Oceania (*k* = 1) and sub-Saharan Africa (*k* = 1). A minimal sample number of six from a particular geographical region would qualify its inclusion in the moderator analysis. Most samples included school age adolescents (*k* = 56) and only seven samples included children under the age of 10 years. Half of the samples used maternal education as their study factor (*k* = 32), while the others assessed education of fathers, caregivers, wage earners or the highest education in the household or between parents. In total, 47 samples incorporated ISCED 3 or equivalent in their classification of parental education, therefore allowing us to perform secondary comparisons as detailed in ‘Methods’ section. Among the 63 samples included, 39 investigated suicidal thought/ideation as one of their primary outcomes, and 46 investigated suicidal attempt/death, 21 studied both. Most samples assessed these outcomes through questionnaires (*k* = 40), and the majority derived information regarding suicidal behaviours directly from the participants (*k* = 49). Among the samples included, 34 originally reported adjusted ORs, ORs or beta coefficients, while 29 reported cross-tabulated data. The results of the risk of bias assessment are presented in the online Supplementary material (Tables S1–S3). Among the 39 samples that reported an association between parental education and suicidal ideation, 62% (*k* = 24) fell into the high-risk category, 36% (*k* = 14) were rated as of some concerns and only 2% (*k* = 1) were rated as low risk. On the other hand, of the 46 samples that evaluated suicidal attempt, 59% were rated as low or of some concern (*k* = 6 and 21), while 41% (*k* = 19) were rated as high risk.

**Fig. 2.**
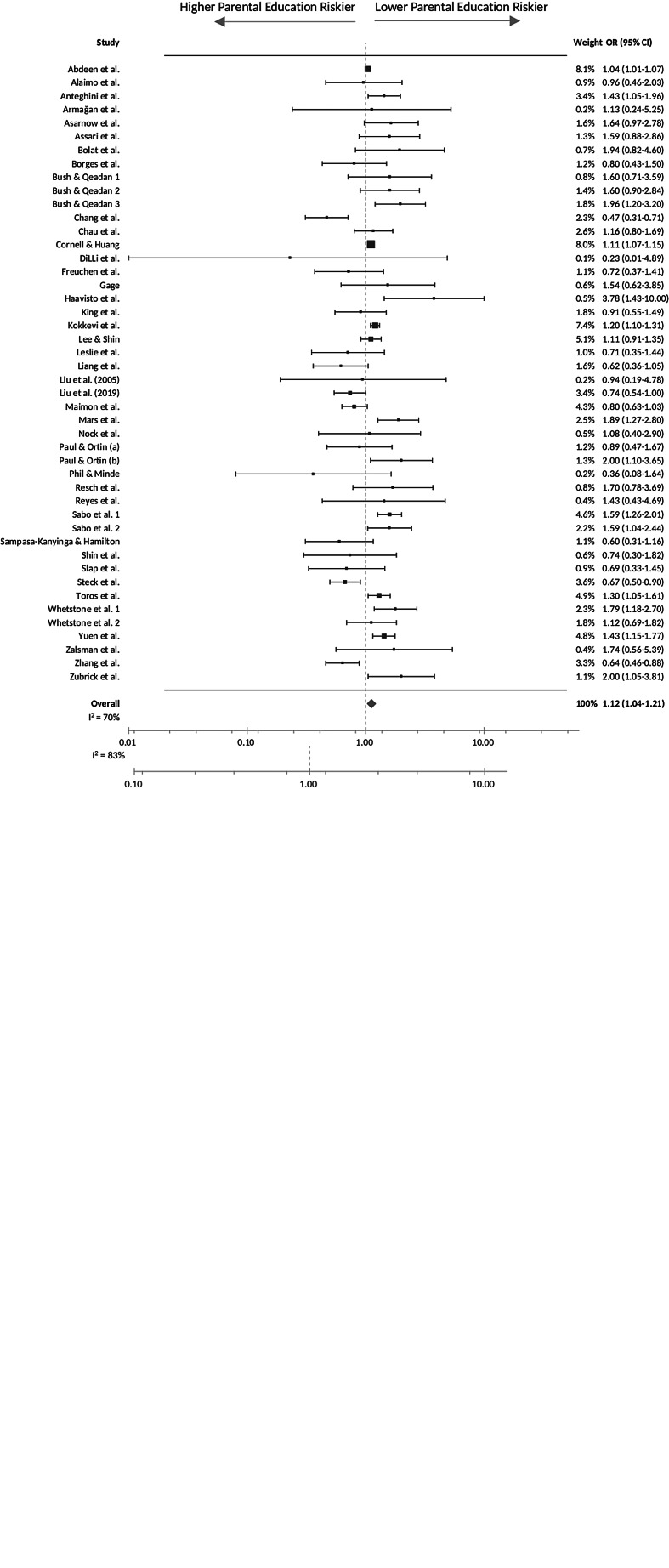
(*a*) Primary analysis: forest plot of the association between parental education and youth suicidal ideation. (*b*) Primary analysis: forest plot of the association between parental education and youth suicidal attempts.

For the purpose of evaluating the overall effect of parental educational on youth suicidal behaviours, in the primary meta-analyses we used ORs of the lowest parental education level defined in each study with the highest parental educational level as the reference wherever possible, to estimate effect sizes. Pooled effect sizes indicate the risk or likelihood for youth suicidal behaviours for youths with the lower educated parents. Figures 2*a* and 2*b* summarise the pooled ORs for suicidal ideation and suicidal attempt. The pooled results reveal a small, but positive association between lower parental education and youth suicidal attempts (OR = 1.12, 95% CI = 1.04–1.21), but not suicidal ideation (OR = 1.05, 95% CI = 0.98–1.12). The heterogeneity ranged from moderate (*I*^2^ = 70% for suicidal attempt) to substantial (*I*^2^ = 83% for suicidal ideation), indicating the need for moderator analyses (Table 2). These showed that geographical region (*p* = 0.008) and country income level (*p* = 0.02) were significant moderators of the direction and strength of the association between lower parental education and youth suicidal attempts and ideation. In particular, lower parental education was associated with an increased risk of youth suicidal attempts for studies conducted in Northern America (OR = 1.26, 95% CI = 1.10–1.45), but such association was reversed in studies conducted in Eastern and South-Eastern Asia, where higher parental education was associated with an increased risk of youth suicide attempts (OR = 0.72, 95% CI = 0.54–0.96). In addition, lower parental education was only associated with an increased risk of youth suicidal ideation in HICs (OR = 1.14, 95% = 1.05–1.25), and the association was absent in studies conducted in low- and middle-income countries (LMICs) (OR = 0.91, 95% CI = 0.77–1.08). Egger's regression test indicated no significant publication bias for both outcomes. The funnel plots also showed no notable asymmetries (online Supplementary Figs S1A and S1B).

**Table 2.** Univariate moderator analysis of the relationship between parental education and youth suicidal behaviours

Outcome	Moderator	*k*	*N*	Effect size analysis				Heterogeneity analysis
				*b*	OR	95% CI	*p*	*I*^2^ (%)
*Suicidal ideation*		39	241 047					
	*Sample type*						0.47	
	Community	38	230 687		1.05	0.98–1.13		82.70
	Clinical	1	360		1.47	0.60–3.61		N/A
	*% Female (continuous)*	35	226 925	0.05			0.66	
	*Study design*						0.37	
	Cross-sectional	38	240 809		1.04	0.97–1.12		83.00
	Longitudinal	1	238		1.41	0.74–2.69		N/A
	*Country income level*						0.02*	
	High	26	185 923		1.14	1.05–1.25		71.60
	Low-middle	13	55 124		0.91	0.77–1.08		85.50
	*Geographical region*						0.35	
	Europe and Northern America	18	99 406		1.14	1.00–1.29		78.10
	Eastern and South-Eastern Asia	13	122 833		0.96	0.81–1.28		87.80
	*Outcome assessment*						0.15	
	Questionnaire	28	218 438		1.06	0.99–1.15		86.80
	Other	11	22 609		0.93	0.80–1.09		6.40
	*Outcome assessment subject*						0.23	
	Child	31	231 504		1.04	0.97–1.12		85.90
	Other	8	9543		1.24	0.93–1.66		0.00
	*Timeframe*						0.76	
	Lifetime	15	29 210		1.08	0.91–1.30		83.50
	Other	24	211 837		1.05	0.96–1.15		78.50
	*Risk of bias*						0.05	
	Low	1	1090		1.59	0.89–2.84		N/A
	Some concern	14	85 425		0.89	0.73–1.09		86.30
	High	24	154 532		1.14	1.03–1.25		80.40
*Suicidal attempt*		46	2 704 716					
	*Sample type*						0.52	
	Community	41	2 701 947		1.12	1.03–1.20		72.50
	Clinical	5	2769		1.27	0.86–1.87		17.30
	*% Female (continuous)*	37	2 634 597	0.12			0.35	
	*Study design*						0.001	
	Cross-Sectional	45	309 039		1.14	1.06–1.23		68.90
	Longitudinal	1	2 395 677		0.67	0.50–0.90		N/A
	*Country income level*						0.07	
	High	34	2 648 092		1.18	1.09–1.28		68.50
	Low-middle	12	56 624		0.90	0.67–1.20		74.00
	*Geographical region*						0.008*	
	Europe	6	2 448 061		1.12	0.80–1.56		77.70
	Northern America	22	115 831		1.26	1.10–1.45		64.60
	Eastern and South-Eastern Asia	7	118 761		0.72	0.54–0.96		69.40
	Western Asia	6	11 151		1.17	0.96–1.41		37.70
	*Outcome assessment*						0.23	
	Questionnaire	27	278 677		1.16	1.07–1.26		77.40
	Other	19	2 426 039		1.02	0.83–1.25		42.30
	*Outcome assessment subject*						0.95	
	Child	37	299 239		1.13	1.05–1.22		71.90
	Other	9	2 405 477		1.12	0.78–1.60		64.50
	*Timeframe*						0.30	
	Lifetime	20	2 481 278		1.17	1.02–1.35		62.30
	Other	26	223 438		1.07	0.98–1.18		72.60
	*Risk of bias*						0.41	
	Low	6	3194		1.13	0.79–1.62		45.20
	Some concern	21	118 727		1.22	1.04–1.43		66.30
	High	19	2 582 795		1.06	0.94–1.20		74.90

*p < 0.05.

**Fig. 3.**
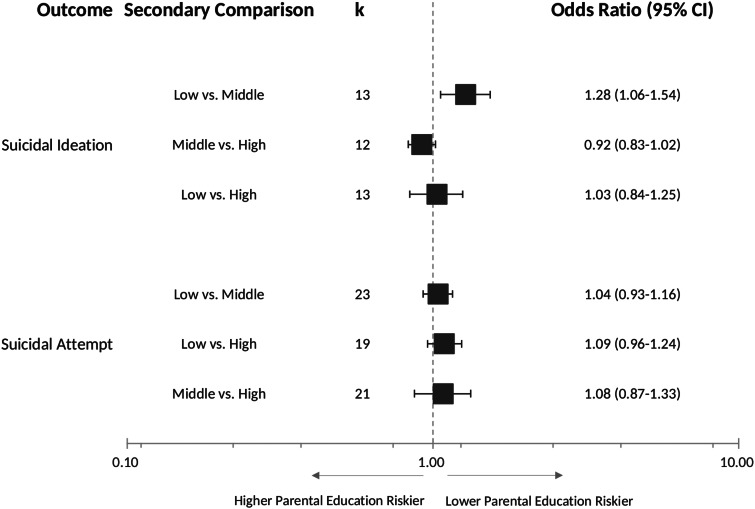
Secondary analysis: forest plot of the associations between lower parental education and youth suicidal behaviours across parental education level subgroups.

A total of 47 samples incorporated ISCED 3 or equivalent in their classification of parental education. These studies were selected for the secondary analyses, in which we evaluated the relationship between lower parental education and youth suicidal behaviours across three parental education level subgroups (low, middle and high). Pooled results showed an increase in risk for suicidal ideation in youths of parents with low education level compared to those of parents with middle-educational level (*k* = 13, OR = 1.28, 95% CI = 1.06–1.54) (Fig. 3).

## Discussion

Our main finding is that lower parental education is significantly associated with a small increase in the risk of youth suicidal attempts. Furthermore, we found that having parents with a low education level (below ISCED 3) is associated with a higher risk of suicidal ideation than having parents with a middle-education level (equals to ISCED 3). Finally, we also found that the association between parental education and youth suicidal behaviours is moderated by both geographic region and country income level. Specifically, lower parental education is associated with an increased risk of youth suicidal ideation and attempts in studies conducted in HICs and Northern America, respectively, while the opposite is true for studies conducted in Eastern and South-Eastern Asia, where higher parental education appears to be associated with a higher risk of youth suicidal attempts.

Our first finding is consistent with reports from an older systematic review conducted by Evans *et al*. (2004), which reported that among family socioeconomic characteristics, lower parental educational level and worries for family finance were the only factors associated with an increased risk of adolescent suicidality. Multiple potential pathways have been proposed to mediate the association between higher parental education level and more favourable youth health outcomes. For instance, several studies conducted in the West support that higher parental education is associated with better parent–child interaction (Zayas *et al*., 2000), more positive parenting (Carr and Pike, 2012), healthier lifestyle (Jablonska *et al*., 2012) and increased resource buffering against stressful life events and supporting children's problem solving (Reiss *et al*., 2019). Higher parental education could also be indicative of a broad social and economic positive influence on the home environment, as higher education could give access to higher earnings and more affluent living (Lindeboom *et al*., 2009). Higher education could also enable parents to better recognise problematic issues in adolescents via stronger mental health literacy and access to sources of support (Villatoro *et al*., 2018). All of the above could potentially help promote child and adolescents' well-being and better mental health. In line with this, our first finding supports a possible protective role of higher parental education against youth suicidal attempts.

In contrast, we found no association between lower parental education and youth suicidal ideation in the primary analysis, although such an association became evident in a secondary analysis across education level subgroups, where low education levels were associated with an increased risk of suicidal ideation compared to middle-education levels. The fact that lower parental education was associated with an increased risk of youth suicidal attempts but not with a risk of suicidal ideation in our primary analysis somewhat echoes an observation previously made by Kapi *et al*. (2007), who suggested that family SES could be more closely related to externalising behaviours rather than internalising domains of adolescent psychopathology. Also, 90% of participants included in studies of suicidal ideation were in their middle to late adolescence, and some authors have suggested that the influence of family SES on youth mental health outcomes could diminish with age (Bøe *et al*., 2012).

Taken together, the findings of our primary and secondary analyses suggest that the relationship between parental education and youth suicidal ideation might not be linear. Different parental educational milestones may have different effects on this particular outcome, as our secondary analyses showed youths with parents who completed high school had a relatively lower risk of disclosing suicidal ideation compared to those whose parents did not acquire a high school diploma. In contrast, parental education higher than high school was no longer associated with such reduced risk, suggesting that other factors might counteract a potential protective effect of education.

The relevance of factors other than parental education alone is supported by our finding that geographical region and country income level moderated the relationship between parental education and youth suicidal behaviours. This finding suggests that cultural, psychosocial, economical contexts and possibly biological factors, could play a significant role in this particular association. Previous evidence has suggested that contextual differences could affect the relationship between parental education and youth's well-being (Assari *et al*., 2018). When studying the influence of parental education, it is vital to take into account contextual factors such as politics, racial compositions, societal attitudes, neighbourhood characteristics, in which families are embedded, as the effect of socioeconomic indicators is complex and can vary across different contexts (Assari *et al*., 2018). For instance, while high-parental education may be linked to positive and less harsh parenting styles in Western cultures, it has also been associated with higher academic expectations and performance stress in Asian cultures, particularly Chinese (Chang *et al*., 2017). Meanwhile, social expectations and academic pressure to excel are risk factors shared among youths in Asian countries, and prior research has already highlighted that differences in patterns of suicide between East Asia and the West merit further attention (Kwak and Ickovics, 2019).

Similarly, previous literature has also indicated that cultural and social differences between LMICs and HICs could play a role in the presentation and course of youth self-injurious behaviours (Aggarwal *et al*., 2017). The role of parental education in child health outcomes has become more attenuated over recent decades in low-resource settings as reported by a recent study (Karlsson *et al*., 2019). Our findings are especially important in light of the fact that 78% of all self-imposed lethal acts occur in LMICs, while the vast majority of research concerning youth suicide is based on populations living in North America and in European countries (Kim, 2019). Our results highlight the importance of investigating context-specific risk and protective factors for youth suicidality, as data informing country and regional variation are urgently warranted to identify modifiable risk factors and to inform differential service needs globally (Biswas *et al*., 2020).

Nevertheless, our findings should be interpreted with caution in view of some important limitations. For example, moderate to substantial heterogeneity was present in the studies included in the primary analyses. Despite our extensive efforts to explore the sources, we could identify only some of the many possible moderators. Residual differences between studies could be related to sample characteristics, study design, and definitions and classifications of parental education. In addition, the qualitative assessment revealed that several studies had medium to high risk of bias. This was mainly due to suboptimal practices in exposure ascertainment and outcome assessment, since most studies applied self-administered questionnaires to participants. Also, the cross-sectional nature of most of the data included did not make it possible to conclude whether and how parental education is directly or indirectly associated with youth suicidal behaviours. Finally, the studies included in the meta-analysis varied widely in sample size, with one single study contributing to over 85% of the total participant numbers (Steck *et al*., 2018). However, this study was not overly represented in the synthesis results as it investigated youth suicide death rather than suicidal ideation or attempts. With a much lower prevalence rate, the precision of the study's estimated effect size was reduced despite having a large sample size, which attenuated the study's weight in the random effects model.

On the other hand, the present study also has several strengths. First, we believe that this is the first study to have systematically assessed the effect of parental education as an independent variable in youth suicidal behaviours. As noted in previous research, different indicators of family SES could affect health outcomes through different pathways, and therefore should not be combined (Padilla-Moledo *et al*., 2016). Second, by considering suicidal ideation and attempts separately, we show that these two components of suicidal behaviours, although highly correlated, could in fact have different risk profiles and require different preventive and intervention strategies. Third, our secondary analyses suggest that any effect may not follow a ‘dose-dependent’ pattern. Fourth and last, our results show how critical it is to acknowledge the between-context variation in the association between parental education and youth mental health outcomes.

In conclusion, the present meta-analysis offers a comprehensive synthesis of existing evidence on the relationship between parental education and youth suicidal behaviours, notwithstanding the high heterogeneity of the studies included. In general, our findings provide initial evidence of an association between lower parental education and increased risk of youth suicidal attempt. In addition, the findings suggest that this association may differ across different geographical and economical contexts, possibly related to cultural, psychosocial and/or biological factors. This indicates that it is crucial for future research to gather more evidence on the determinants of youth suicidal behaviours across the global setting. Furthermore, it highlights the importance of taking into account the cultural as well as the familial context in the clinical management of youth suicidal behaviour in our increasingly multicultural societies.

## Supplementary material

For supplementary material accompanying this paper visit http://doi.org/10.1017/S204579602200004X. click here to view supplementary material

## Data

All data used in the systematic review and meta-analyses can be found in the included studies. Extracted data by the authors can be found in the online Supplementary materials.
