# Supplementary material for: Parental education and youth suicidal behaviours: a systematic review and meta-analysis
Source: Epidemiol Psychiatr Sci. 2022 Mar 30;31:e19. doi: 10.1017/S204579602200004X (PMC8967699; doi:10.1017/S204579602200004X)
Supplement: Supplementary file 1 [file S204579602200004Xsup001.zip › S204579602200004Xsup004.docx]

**Table S1.** **Quality assessment of the included cross-sectional studies according to Newcastle-Ottawa Scale**

| **Study** | **Selection** | | | | **Comparability** | **Outcome** | | **Total score** |
| --- | --- | --- | --- | --- | --- | --- | --- | --- |
|  | **Representative sample** | **Adequate sample size** | **Non-respondents** | **Ascertainment of exposure** | **Based on design or analysis** | **Assessment of outcome** | **Statistical test** |  |
| Abdeen et al. | + | - | - | - | ++ | - | + | 4 |
| Alaimo et al. | + | - | - | + | + | + | + | 5 |
| Allen & Goldman-Mellor | + | - | + | + | + | + | + | 6 |
| Amit et al. | + | - | + | + | + | + | + | 6 |
| Anteghini et al. | + | - | - | - | + | - | + | 3 |
| Assari et al. | + | - | - | + | ++ | + | + | 6 |
| Beattie et al. | + | - | - | - | + | - | + | 3 |
| Borges et al. | + | - | - | + | + | + | + | 5 |
| Bush & Qeadan | + | - | + | - | ++ | - | + | 5 |
| Chang et al. | + | - | + | - | ++ | - | + | 5 |
| Chau et al. | + | - | + | - | ++ | - | + | 5 |
| Chen et al. | - | - | - | - | + | - | + | 2 |
| Cornell & Huang | + | - | + | - | ++ | - | + | 5 |
| Franić et al. | + | - | - | - | + | - | + | 3 |
| Gage | + | - | - | - | ++ | - | + | 4 |
| Kim et al. | + | - | - | - | ++ | - | + | 4 |
| King et al. | + | - | + | - | + | - | + | 4 |
| Kokkevi et al. | + | - | - | - | + | - | + | 3 |
| Kovess-Masfety et al. | + | - | - | - | ++ | - | + | 4 |
| Lee & Shin | + | - | + | - | + | - | + | 4 |
| Leslie et al. | + | - | - | + | + | + | + | 5 |
| Liang et al. | + | - | + | - | + | - | + | 4 |
| Liu & Sun | + | - | - | - | + | - | + | 3 |
| Liu et al. (2019) | + | - | - | - | ++ | - | + | 4 |
| Lu et al. | + | - | - | - | ++ | - | + | 4 |
| Martin et al. | +- | - | - | + | + | + | + | 4 |
| Min et al. | + | - | - | - | + | - | + | 3 |
| Nock et al. | + | - | - | + | ++ | + | + | 6 |
| Peter et al. | + | - | - | - | ++ | - | + | 4 |
| Phil & Minde | - | - | - | - | + | - | + | 2 |
| Resch et al. | + | - | - | + | + | + | + | 5 |
| Sabo et al. | + | - | - | - | ++ | - | + | 4 |
| Sampasa-Kanyinga & Hamilton | + | - | + | - | ++ | - | + | 5 |
| Slap et al. | + | - | - | + | + | + | + | 5 |
| Toros et al. | + | + | + | - | + | - | + | 5 |
| Tran et al. | + | - | + | - | ++ | - | + | 5 |
| Wang et al. | + | - | - | - | + | - | + | 3 |
| Whetstone et al. | + | - | - | - | ++ | - | + | 4 |
| Xiao et al. | + | + | + | - | ++ | - | + | 6 |
| Yuen et al. | + | - | + | - | ++ | - | + | 5 |
| Zalsman et al. | + | - | + | + | + | + | + | 6 |
| Zhang et al. | + | - | - | - | + | - | + | 3 |
| Zubrick et al. | + | - | + | - | + | - | + | 4 |
