# Supplementary material for: Parental education and youth suicidal behaviours: a systematic review and meta-analysis
Source: Epidemiol Psychiatr Sci. 2022 Mar 30;31:e19. doi: 10.1017/S204579602200004X (PMC8967699; doi:10.1017/S204579602200004X)
Supplement: Supplementary file 1 [file S204579602200004Xsup001.zip › S204579602200004Xsup005.docx]

**Table S2.** **Quality assessment of the included case-control studies according to Newcastle-Ottawa Scale**

| **Study** | **Selection** | | | | **Comparability** | **Exposure** | | | **Total score** |
| --- | --- | --- | --- | --- | --- | --- | --- | --- | --- |
|  | **Adequate Case Definition** | **Case representative** | **Selection of controls** | **Definition of Controls** | **Based on design or analysis** | **Ascertainment of exposure** | **Same method for cases and controls** | **Non-response rate** |  |
| Armağan et al. | + | + | - | + | + | + | + | + | 7 |
| Bolat et al. | + | + | - | + | + | + | + | + | 7 |
| DiLLi et al. | + | + | - | + | + | + | + | - | 6 |
| Freuchen et al. | - | + | - | - | + | + | - | - | 3 |
| Liu et al. (2005) | - | - | + | - | + | - | - | + | 3 |
