# Supplementary material for: Parental education and youth suicidal behaviours: a systematic review and meta-analysis
Source: Epidemiol Psychiatr Sci. 2022 Mar 30;31:e19. doi: 10.1017/S204579602200004X (PMC8967699; doi:10.1017/S204579602200004X)
Supplement: Supplementary file 1 [file S204579602200004Xsup001.zip › S204579602200004Xsup006.docx]

**Table S3. Quality assessment of the included cohort studies according to Newcastle-Ottawa Scale**

| **Study** | **Selection** | | | | **Comparability** | **Outcome** | | | **Total score** |
| --- | --- | --- | --- | --- | --- | --- | --- | --- | --- |
|  | **Representative exposed cohort** | **Selection of non-exposed cohort** | **Ascertainment of exposure** | **Outcome not present at start of study** | **Based on design or analysis** | **Assessment of outcome** | **Follow-up time** | **Adequate follow-up** |  |
| Asarnow et al. | + | + | + | + | + | + | N/A | + | 7 |
| Chiu et al. | + | + | - | - | + | - | N/A | + | 4 |
| Haavisto et al. | + | + | - | - | ++ | - | N/A | + | 5 |
| Maimon et al. | + | + | + | - | ++ | + | N/A | + | 7 |
| Mars et al. | + | + | - | - | ++ | - | N/A | + | 5 |
| Oppenheimer et al. | + | + | - | + | + | + | N/A | + | 6 |
| Paul & Ortin (2019a) | + | + | + | + | + | + | N/A | + | 7 |
| Paul & Ortin (2019b) | + | + | + | - | + | + | N/A | + | 6 |
| Reyes et al. | + | + | + | - | ++ | + | N/A | + | 7 |
| Shin et al. | - | + | - | + | ++ | - | N/A | - | 4 |
| Steck et al. | + | + | - | - | + | - | N/A | + | 4 |
